# Supplementary material for: Do pride and shame track the evaluative psychology of audiences? Preregistered replications of Sznycer et al. (2016, 2017)
Source: R Soc Open Sci. 2020 May 13;7(5):191922. doi: 10.1098/rsos.191922 (PMC7277259; doi:10.1098/rsos.191922)
Supplement: Initial Replication Bayes Factor Analysis [file rsos191922supp1.docx]

# Supplementary Materials – Part 1

## Initial Replication Bayes Factor Analysis from Study 1

The initial calculation yielded BF_10_ (replication | original) = 1.39, failing to exceed 3 and providing inconclusive evidence for replication success, a notable departure from replication success observed with the other two criteria.

Upon inspection, the discrepancy between the EU-BF and the other measures of replication success appears to be due to a problem in how the initial EU-BF was computed. Note that ${BF}_{10}$ (original + replication) combined the participants from the replication and original datasets, but when data were averaged for each item, the same number of pride-valuation item pairs (n = 25) were generated as ${BF}_{10}$ (original). The replication Bayesian analysis assumes the replication contributes more data to the combined data set, but because the correlation was over items and not participants, the analysis was mostly insensitive to additional data. In the interest of full disclosure and as a cautionary note to others attempting similar analyses over items instead of participants, we have reported the EU-BF analysis above even though it is inappropriate. Because this initial replication Bayes Factor analysis was invalid, we took the two alternative approaches reported in the main text to calculate a valid Bayesian measure of replication success. These two alternatives were more valid than the initial attempt since they more appropriately take into account the additional data provided by the replication.
