# Supplementary material for: Do pride and shame track the evaluative psychology of audiences? Preregistered replications of Sznycer et al. (2016, 2017)
Source: R Soc Open Sci. 2020 May 13;7(5):191922. doi: 10.1098/rsos.191922 (PMC7277259; doi:10.1098/rsos.191922)
Supplement: Priming Analysis [file rsos191922supp2.docx]

# Supplementary Materials – Part 2

**Priming-Control Analyses for Study 1 – Pride**

To reanalyze the data from Study 1, we excluded participants who completed the Shame Study prior to the Pride Study.

### Is the correlation between pride and valuation significantly different from zero and in the same direction as in the original study?

Yes. For each scenario, we calculated the mean pride ratings provided by participants in the pride condition, and the mean valuation ratings provided by participants in the audience condition. The pride means and the valuation means were positively correlated, *r*(23) = 0.81, *p* < 0.001, 95% CI [0.60, 0.91].

**Is the effect size different from zero and not different from the original effect size?**

Using *r* as a measure of effect size, the 95% confidence interval from the replication study, 95% CI [0.60, 0.91], was not consistent with an effect size of zero. Second, the *r* from the replication study, *r*(23) = 0.81, fell within the 95% confidence interval from the original study, 95% CI [0.69, 0.93].

**Is the replication Bayes Factors greater than 3 and in favor of the alternative hypothesis relative to the null hypothesis?**

Yes. We used the two alternative Bayesian approaches from Study 1 since the initial approach was invalid. On the first alternative approach, we combined the 25 item pairs from the original and replication studies as if they were separate items to calculate $\mathrm{BF}_{10}$(original + replication). Dividing this by BF_10_(original) yielded BF_10_(replication|original) = 7.26 x 10^4^, exceeding a Bayes factor of 3. On the second alternative approach, we generated a posterior for ρ from the original study and used it as the prior for the replication study. The recalculated BF_10_ (replication|original) was 1.12 x 10^5^, which exceeded a Bayes factor of 3. These results are consistent with the frequentist replication analyses, and together provide evidence for replication of the original results

**Priming-Control Analyses for Study 2 – Shame**

To reanalyze the data from Study 2, we excluded participants who completed the Pride Study prior to the Shame Study.

**Is the correlation between shame and devaluation significantly different from zero and in the same direction as in the original study?**

Yes. For each scenario, we calculated the mean shame ratings provided by participants in the shame condition, and the mean devaluation ratings provided by participants in the audience condition. The shame means and the devaluation means were positively correlated, *r*(27) = 0.72, *p* < 0.001, 95% CI [0.48, 0.86].

**Is the effect size different from zero and not different from the original effect size?**

Using *r* as a measure of effect size, the 95% confidence interval from the replication study, 95% CI [0.48, 0.86], was not consistent with an effect size of zero. Second, the *r* from the replication study, *r*(27) = 0.72, fell within the 95% confidence interval from the original study, 95% CI [0.48, 0.86].

**Is the replication Bayes Factors greater than 3 and in favor of the alternative hypothesis relative to the null hypothesis?**

Yes. We used the two alternative Bayesian approaches from Study 1 since the initial approach was invalid. On the first alternative approach, we combined the 29 item pairs from the original and replication studies as if they were separate items to calculate $\mathrm{BF}_{10}$(original + replication). Dividing this by BF_10_(original) yielded BF_10_(replication|original) = 7.27 x 10^3^, exceeding a Bayes factor of 3. On the second alternative approach, we generated a posterior for ρ from the original study and used it as the prior for the replication study. The recalculated BF_10_ (replication|original) was 1.19 x 10^4^, which exceeded a Bayes factor of 3. These results are consistent with the frequentist replication analyses, and together provide evidence for replication of the original results.
